# Pro-inflammatory activation of primary microglia and macrophages increases 18 kDa translocator protein expression in rodents but not humans

**DOI:** 10.1177/0271678X17710182

**Published:** 2017-05-22

**Authors:** David R Owen, Nehal Narayan, Lisa Wells, Luke Healy, Erica Smyth, Eugenii A Rabiner, Dylan Galloway, John B Williams, Joshua Lehr, Harpreet Mandhair, Laura AN Peferoen, Peter C Taylor, Sandra Amor, Jack P Antel, Paul M Matthews, Craig S Moore

**Affiliations:** 1 Division of Brain Sciences, Department of Medicine Hammersmith Hospital, Imperial College London, London, UK; 2 Nuffield Department of Orthopaedics, Rheumatology & Musculoskeletal Sciences, Botnar Research Centre, University of Oxford, Oxford, UK; 3 Imanova Centre for Imaging Science, Hammersmith Hospital, London, UK; 4 Neuroimmunology Unit, Montreal Neurological Institute, McGill University, Montreal, Quebec, Canada; 5 Centre for Neuroimaging Sciences, King’s College, London, UK; 6 Division of Biomedical Sciences, Faculty of Medicine, Memorial University of Newfoundland, St. John’s, Newfoundland; 7 Pathology Department, VU Medical Centre, VU University of Amsterdam, The Netherlands; 8 Neuroimmunology Unit, Blizard Institute, Barts and the London School of medicine & Dentistry Queen Mary University of London, UK; 9 UK Dementia Research Institute, Imperial College London, London, UK

**Keywords:** Positron emission tomography, microglia, macrophages, inflammation, neurodegeneration

## Abstract

The 18kDa Translocator Protein (TSPO) is the most commonly used tissue-specific marker of inflammation in positron emission tomography (PET) studies. It is expressed in myeloid cells such as microglia and macrophages, and in rodent myeloid cells expression increases with cellular activation. We assessed the effect of myeloid cell activation on *TSPO* gene expression in both primary human and rodent microglia and macrophages in vitro, and also measured TSPO radioligand binding with ^3^H-PBR28 in primary human macrophages. As observed previously, we found that *TSPO* expression increases (∼9-fold) in rodent-derived macrophages and microglia upon pro-inflammatory stimulation. However, *TSPO* expression does not increase with classical pro-inflammatory activation in primary human microglia (fold change 0.85 [95% CI 0.58–1.12], *p* = 0.47). In contrast, pro-inflammatory activation of human monocyte-derived macrophages is associated with a reduction of both *TSPO* gene expression (fold change 0.60 [95% CI 0.45–0.74], *p* = 0.02) and TSPO binding site abundance (fold change 0.61 [95% CI 0.49–0.73], *p* < 0.0001). These findings have important implications for understanding the biology of TSPO in activated macrophages and microglia in humans. They are also clinically relevant for the interpretation of PET studies using TSPO targeting radioligands, as they suggest changes in TSPO expression may reflect microglial and macrophage density rather than activation phenotype.

## Introduction

Microglia are resident immune cells of the central nervous system (CNS).^1^ In response to endogenous or exogenous danger signals such as neuronal injury or bacterial lipopolysaccharide (LPS), microglia rapidly activate.^1^ This involves a myriad of changes to morphology, metabolism, surface marker expression and cytokine release, but the range of activation phenotypes is broad and microglia can generate both pro-inflammatory and reparative effector functions.^2,3^ Dysregulated or inappropriate microglial activation is thought to contribute to many neurodegenerative diseases.^4^ Modulating microglial phenotype has therefore been hypothesised as a potential therapeutic strategy for diseases such as Alzheimer’s and Parkinson’s Disease.^4^

The 18 kDa Translocator Protein (TSPO) is an outer mitochondrial membrane protein which is highly conserved across eukaryotic kingdoms, and an analogue is also present on the cell membrane of prokaryotes.^5^ It is found in most cell types but its expression is high in phagocytic cells of the immune system such as microglia and macrophages.^5^ A substantial body of literature has documented increases in TSPO expression in a range of human diseases in post mortem brain tissue and in animal models of neuroinflammation and neurodegeneration.^6–9^ For this reason, TSPO is the most commonly used marker of inflammation in positron emission tomography (PET) studies in clinical populations.^10^ Furthermore, preclinical experiments show that TSPO ligands provide protection in animal models of neuroinflammation and degeneration.^11–20^ In vitro studies suggest that the mechanism behind this protection may be via a beneficial shift in microglial phenotype.^13,15,21–24^ While the precise function of TSPO is unknown, preliminary studies suggest TSPO may alter myeloid phenotype by modulating the increases in reactive oxygen species (ROS) production and bioenergetics shifts that accompany myeloid cellular activation.^15,25^ In vitro, both mRNA and protein expression of TSPO increase substantially following activation (e.g. with LPS) in rodent myeloid cells.^13,15,21,26^ However, whether TSPO expression alters with myeloid cell activation in humans is not known.

Here, we assessed and contrasted the effect of myeloid cell activation on *TSPO* gene expression in both primary human and rodent microglia and macrophages in vitro. We also measure TSPO radioligand binding with the TSPO targeting radioligand ^3^H-PBR28^9^ in primary human macrophages. As observed previously, we found that *TSPO* expression increases in rodent-derived myeloid cells upon activation. However, an unexpected and novel observation is that *TSPO* expression does not increase with classical pro-inflammatory activation in primary human microglia. In contrast, pro-inflammatory activation of human monocyte-derived macrophages is associated with a reduction of both *TSPO* gene expression and TSPO binding site abundance. These findings have important implications for understanding the biology of TSPO in activated macrophages and microglia in humans. They are also clinically relevant for the interpretation of PET studies using TSPO targeting radioligands.

## Materials and methods

### Human microglia isolation (temporal lobe biopsies and foetal samples) and culture

Adult microglia were isolated from a mixture of white and gray matter of temporal lobe brain tissue (n = 18), from patients undergoing surgery for intractable epilepsy not related to tumours in accordance with the guidelines set by the Biomedical Ethics Unit of McGill University and approved under reference ANTJ2001/1. All experiments were conducted in accordance with the Helsinki Declaration. Written informed consent was obtained from all subjects. There was no gender bias. The tissue provided was outside of the suspected focal site of epilepsy-related pathology. The surgical brain tissue was processed as described previously.^3,27^ Briefly, tissue was obtained in pieces <1 mm^3^ and treated with DNase (Roche, Nutley, NJ) and trypsin (Invitrogen, Carlsbad, CA) for 30 min at 37℃. Following dissociation through a nylon mesh, the cell suspension was separated on a 30% Percoll gradient (GE Healthcare, Piscataway, NJ) at 31,000 *g* for 30 min. Glial cells (oligodendrocytes and microglia) were collected from underneath the myelin layer, washed and then plated in tissue-culture-treated vessels. Floating oligodendrocytes were washed off on the subsequent day and the remaining adherent microglia were collected with trypsin and 2 mM EDTA (Sigma-Aldrich). Cells were plated at 1 × 10^5^ cells mL^−1^ in minimum essential medium (Sigma) containing 5% foetal bovine serum (FBS), 100 U mL^−1^ penicillin and 100 µg mL^−1^ streptomycin and 2 mM glutamine (all from Invitrogen). The proportion of microglia in the culture, determined using CD11c staining by flow cytometry, was ≥90%.^3,28^

Human foetal CNS tissue (cerebral hemispheres, n = 8) was obtained from the human foetal tissue repository (Albert Einstein College of Medicine, Bronx, NY), following approved institutional and Canadian Institutes for Health Research guidelines. Cells were isolated as previously described.^29^ Briefly, brain tissue (gestational age 14–20 weeks) was minced and treated with DNase/trypsin. Tissue was then dissociated through a nylon mesh and cells were plated at 6 × 10^6^ cells mL^−1^ in high glucose DMEM with 5% FBS, 100 U mL^−1^ penicillin and 100 µg mL^−1^ streptomycin and 2 mM glutamine (all from Invitrogen). After 10–14 days in culture, floating microglia were harvested and plated at 1 × 10^5^ cells mL^−1^.

For pro-inflammatory stimulation, cells were treated with human granulocyte-macrophage colony-stimulating factor (GM-CSF, 5 ng mL^−1^, PeproTech, Rocky Hill, NJ) for five days followed by 1 h stimulation with IFNγ (20 ng mL^−1^, Invitrogen) and 48 h stimulation with lipopolysaccharide LPS (serotype 0127:B8, 100 ng mL^−1^, Sigma). For stimulation towards a reparative phenotype, cells were treated with macrophage colony-stimulating factor (M-CSF, 25 ng mL^−1^, PeproTech) for five days followed by 48 h stimulation with IL-4 (20 ng mL^−1^, Invitrogen) and IL-13 (20 ng mL^−1^, PeproTech). Both stimulation protocols reliably generate the respective phenotypes.^3,28,30^ To confirm pro-inflammatory activation, TNF-α production or TNF-α gene expression was measured by ELISA (BD Biosciences, as per manufacturer’s protocol) or qPCR (methods below) respectively in a subset of samples (12/26). In all samples tested for TNF-α gene expression, TNF-α was at least 8-fold higher in IFNγ/LPS-stimulated cells relative to unstimulated cells. In all samples tested for TNF-α production, TNF-α was greater than 4000 ng mL^−1^ in all IFN-γ/LPS stimulated cells and undetected in unstimulated cells.

### Human monocyte isolation and macrophage generation

For quantification of *TSPO* gene expression, venous blood was collected from healthy volunteers (age range 21–53 years, no gender bias) under a protocol approved by the institutional review board of McGill University (ANTJ2001/1). Written informed consent was obtained from all subjects. To isolate monocytes, peripheral blood mononuclear cells (PBMCs) were separated on a Ficoll density gradient (GE Healthcare). CD14+ monocytes were positively selected by MACS using anti-CD14 microbeads (Miltenyi Biotec, Auburn, CA), then plated at 5 × 10^5^ cells mL^−1^ in 24- or 6-well plates in RPMI with 10% FBS, 100 U mL^−1^ penicillin and 100 µg mL^−1^ streptomycin and 2 mM glutamine (all from Invitrogen). To generate monocyte-derived macrophages and induce a pro-inflammatory phenotype, monocytes were treated for five days with human recombinant GM-CSF (5 ng mL^−1^) and then activated for 1 h with IFN-γ (20 ng mL^−1^) and 48 h with LPS (100 ng mL^−1^). To induce a reparative phenotype, monocytes were treated for five days with M-CSF (25 ng mL^−1^) and then activated for 48 h with IL-4 (20 ng mL^−1^) and IL-13 (20 ng mL^−1^). To confirm pro-inflammatory activation, TNF-α production or TNF-α gene expression was measured by ELISA (BD Biosciences, as per manufacturer’s protocol) or qPCR (methods below) respectively in a subset of samples (12/15). In all samples tested for TNF-α gene expression, TNF-α was at least 6-fold higher in IFNγ/LPS-stimulated cells relative to unstimulated cells. In all samples tested for TNF-α production, TNF-α was greater than 1500 ng mL^−1^ in all IFN-γ/LPS-stimulated cells and undetected in unstimulated cells.

For quantification of TSPO binding signal, very large cell numbers are required and therefore peripheral blood monocytes were isolated from blood cones of healthy donors purchased from the National Blood Service (Colindale, Edgware, London UK) using density gradient centrifugation, and centrifugal elutriation performed utilizing flow cytometry to isolate fractions of 90% purity monocytes as previously described.^31^ Upon collection of monocyte-rich fractions by elutriation, cells were counted and re-suspended to a density of 1 million cells/ml in RPMI supplemented with 10% endotoxin free heat-inactivated, 100 U/ml penicillin and 100 µg mL^−1^ streptomycin (Lonza, Biowhittaker®, Belgium). Macrophages were then derived from the elutriated monocytes by culturing cells with 100 ng mL^−1^ M-CSF (Peprotech Inc., Rocky hill, NJ, USA) in 24-well cell plates at a concentration of 1 million cells/ml, with differentiation for seven days. Cells were then stimulated with LPS 10 ng mL^−1^, or IL-4 20 ng mL^−1^ (both from Peprotech Inc., Rocky hill, NJ) for 24 h, or media alone. The supernatant was subsequently removed and the cells harvested on ice. To confirm pro-inflammatory activation, TNF-α gene expression was measured by qPCR (methods below) in all samples and was at least 6-fold higher in stimulated cells relative to unstimulated cells.

### Isolation and culture of mouse myeloid cells

All protocols were approved by the animal care committee of Memorial University, Canada. The experiments were conducted in accordance with The Canadian Council on Animal Care CCAC guidelines, and reported in compliance with ARRIVE guidelines. For microglia, mixed glia cultures were derived from P0-P5 C57Bl/6 mouse pups purchased from Charles River and cultured in DMEM supplemented with 10% FBS, 100 U mL^−1^ penicillin and 100 µg mL^−1^ streptomycin and 2 mM glutamine (all from Invitrogen). At confluency, cultures were subjected to mild trypsinization as described.^32^ Following removal of the astrocyte monolayer, microglia were re-plated at a density of 2 × 10^5^ cells mL^−1^ in DMEM + 10% FBS, 100 U mL^−1^ penicillin and 100 µg mL^−1^ streptomycin and 2 mM glutamine (all from Invitrogen) with a 1:1 ratio with astrocyte conditioned media (media collected from confluent rodent astrocytes) prior to stimulation. Microglia were allowed to adhere for at least 24 h before prior to experimentation. For bone marrow-derived macrophages (MDM), bone marrow cells were isolated from the femurs and tibias of 6–12-week-old C57Bl/6 mice (purchased from Charles River). There was no gender bias. Red blood cells were lysed in an ammonium chloride solution, and the remaining bone marrow cells were cultured at 2.5 × 10^6^ cells mL^−1^ in DMEM supplemented with 10% FBS, 100 U mL^−1^ penicillin and 100 µg mL^−1^ streptomycin and 2 mM glutamine (all from Invitrogen) and M-CSF (10 ng mL^−1^). Media was replaced on day 3, cells were treated with LPS (100 ng mL^−1^ L) or vehicle on day 7, and harvested on day 9.

### RNA Isolation, reverse transcription, and real-time PCR

For HAM, FM and MDM used in the *TSPO* gene expression experiments, tissue was stored at −80℃ in TRIzol (Invitrogen) for subsequent total RNA isolation using the Qiagen RNeasy mini kit following manufacturer’s instructions. RNA isolated was treated immediately with DNase (Qiagen, Germantown, MD). Reverse transcription and cDNA generation were performed using random hexaprimers (Roche) and the Moloney murine leukemia virus-RT enzyme (Invitrogen) at 42℃. PCR reaction cycling was performed according to the ABI PRISM 7000 Sequence Detection System default temperature settings (2 min at 50℃, 10 min at 95℃, followed by 40 cycles of 15 s at 95℃, 1 min at 60℃). The housekeeping gene 18 S was used as a reference for normalization of transcript abundance. Fold changes in gene expression were calculated using the ^ΔΔ^*C*_t_ method according to manufacturer’s instructions. For MDM used in the radioligand binding experiments, RNA was isolated using the Total RNA kit E.Z.N.A.™ EaZy Nucleic Acid Isolation (Omega Bio-tek). cDNA was synthesized via reverse transcription utilizing a Veriti™ 96-well Thermal cycler (Applied Biosystems, Life Technologies, Paisley UK). The theromcycler ViiA™ 7 real-time PCR system (Applied Biosystems, Paisley UK) was used to perform real-time quantitative PCR. A spin protocol method was used to extract DNA from peripheral whole blood. A QIAamp DNA Blood mini kit was utilized (QIAGEN, Hilden), and the spin protocol followed for this kit. The TaqMan ® SNP Genotyping Assay (Taqman® SNP Genotyping assay, Applied Biosystems) was utilized to undertake genotyping for the rs6971 SNP on DNA extracted from peripheral whole blood.

### Radioligand binding experiments

[^3^H]PBR28 (N-[[2-(methyloxy)phenyl]methyl]-N-[4-(phenyloxy)-3-pyridinyl]acetamide; specific activity = 82 Ci/mmol; Radioactive concentration = 1.0 mCi mL^−1^) was custom labelled by Tritech, Switzerland. Unlabelled PK11195 was purchased from Sigma, UK. Cells were homogenised in 10 times weight for volume buffer (0.32 mM sucrose, 5 mM Tris Base, 1 mM MgCl_2_, pH 7.4, 4℃). Homogenates were centrifuged (32,000 × *g*, 20 min, 4℃) followed by removal of the supernatant. Pellets were re-suspended in at least 10 times w/v buffer (50 mM Tris Base, 1 mM MgCl_2_, pH 7.4, 4℃) followed by two washes by centrifugation (32,000 × *g*, 20 min, 4℃). Membranes were suspended in buffer (50 mM Tris Base, 1 mM MgCl_2_, pH 7.4, 4℃) and aliquots were stored at −80℃ until use. Aliquots of membrane suspension (containing 40 µg protein) were prepared using assay buffer (50 mM Tris Base, 140 mM NaCl, 1.5 mM MgCl_2_, 5 mM KCl, 1.5 mM CaCl_2_, pH 7.4, 37℃) and incubated with [^3^H]PBR28 at 37℃ in a final volume of 250 µl for 60 min. Eight concentrations of [^3^H]PBR28 were used, ranging from 100 pM to 100 nM. The specific binding component was defined by addition of unlabelled PK11195 (10 μM). Following incubation, assays were terminated via filtration through Whatman GF/C filters, followed by 3 × 1 ml washes with ice-cold wash buffer (50 mM Tris Base, 1.4 mM MgCl_2_, pH 7.4, 4℃). Whatman GF/C filters were pre-incubated with 0.05% polyethyleneimine (60 min) prior to filtration. Scintillation fluid (3 ml/vial, Perkin Elmer Ultima Gold MV) was added and vials counted on a Perkin Elmer Tricarb 2900 liquid scintillation counter. Each point was performed in triplicate. Protein concentrations (µg protein/ml) were determined using the Bicinchoninic acid assay (BCA Kit, Sigma-Aldrich, UK) and absorption read at 562 nm. Saturation data were analysed using the iterative non-linear regression curve fitting software supplied with GraphPad Prism 5.0. The extra sum of squares F test was used to determine whether the parameters B_max_ and K_d_ differ among the data sets.

### Western blotting

Cells were lysed in standard radioimmunoprecipitation assay buffer supplemented with BD Baculogold protease inhibitors (BD Biosciences) and 1 mM Na_3_VO_4_. Protein lysates were then quantified and run on a 10% acrylamide gel by sodium dodecyl sulfate–polyacrylamide gel electrophoresis. Following transfer to polyvinylidene difluoride, membranes were blocked in 5% milk powder, probed with a monoclonal antibody against TSPO (0.1 mg/mL PA5-18565 ThermoFisher, Waltham, MA) and detected with an HRP-goat anti-rabbit antibody (1 mg/mL, 611620 ThermoFisher, Waltham, MA) and ECL Plus reagents (GE Healthcare, Piscataway, NJ). Equal protein loading was confirmed by probing with a monoclonal antibody against beta-actin (1:1000, monoclonal anti-actin, A4700 Sigma-Aldrich, UK) and detected with an HRP-rabbit anti-mouse antibody (1 mg/mL, P0260, Dako, Denmark)

### Statistical analyses

Statistical analyses were performed using Prism 5 (GraphPad Software). Comparisons across groups were analyzed by one-way ANOVA or *t*-tests as appropriate. Probability values of <0.05 were considered to represent statistically significant differences.

## Results

### Basal TSPO gene expression is higher in human monocyte derived macrophages than in microglia

**Figure 1.**
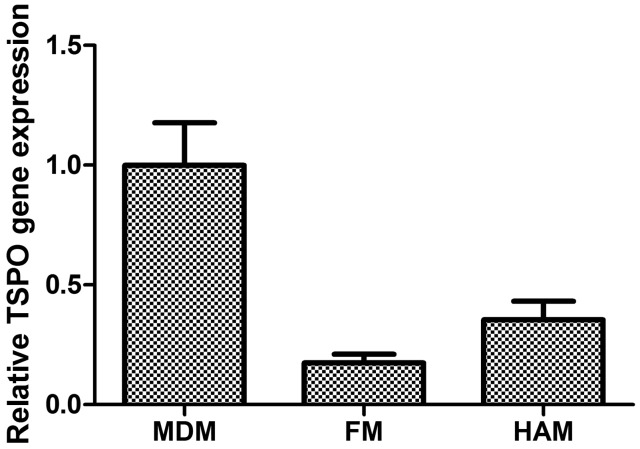
Changes in TSPO gene expression of unstimulated foetal microglia (FM, n = 8) and human adult microglia (HAM, n = 18) relative to expression in monocyte derived macrophages (MDM, n = 15) (ANOVA, *p* < 0.0002).

Under basal conditions, the mean *TSPO* gene expression in human monocyte derived macrophages (MDM) (n = 15) measured by qPCR was used as a reference and compared with human adult microglia (HAM) (n = 18) and with foetal microglia (FM) (n = 8). *TSPO* expression was higher both in MDM relative to HAM (5.72-fold reduction [95% CI 3.86–11.07]) and in MDM relative to FM (2.82-fold reduction [95% CI 1.93–5.27]) (*p* < 0.0002, one-way ANOVA)(Figure 1).

### TSPO gene expression does not increase in activated primary human microglia

**Figure 2.**
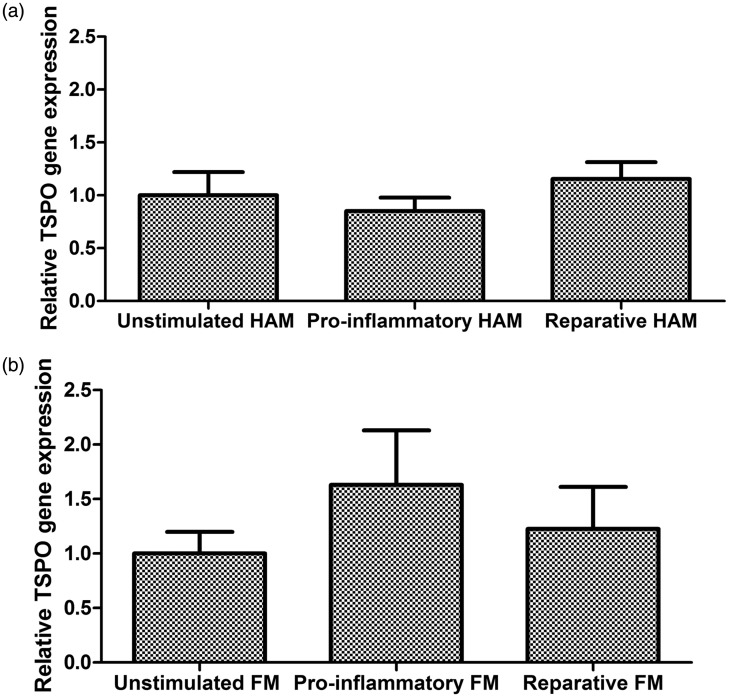
Relative changes in TSPO gene expression in independent cohorts of human microglia. Stimulated conditions are referenced relative to unstimulated conditions. (a) Human adult microglia (n = 18, ANOVA, *p* = 0.47). (b) Foetal microglia (n = 8, ANOVA, *p* = 0.51).

Under stimulated conditions, the mean *TSPO* gene expression in unpolarised cells was used as the reference. With HAM (n = 18), neither IFN-γ/LPS (a classical activation pro-inflammatory stimulus) nor IL4/IL13 (a stimulus of reparative activation) increased gene expression (One way ANOVA, *p* = 0.47, Figure 2). Relative to the unstimulated condition, the fold change in *TSPO* gene expression with IFN-γ/LPS activation was 0.85 [95% CI 0.58–1.12] and with IL4/IL13 activation was 1.16 [95% CI 0.82–1.49]. Comparable results were found with FM (n = 8). Neither the classic pro-inflammatory stimulus nor the reparative stimulus significantly increased *TSPO* gene expression (One way ANOVA, *p* = 0.51; Figure 2). Compared with unstimulated cells, the relative change in IFN-γ/LPS stimulated cells was 1.63 [95% CI 0.45–2.81] and in IL4/IL13 stimulated cells was 1.23 [95% CI 0.32–2.14].

### TSPO gene expression decreases under pro-inflammatory conditions in primary human macrophages

**Figure 3.**
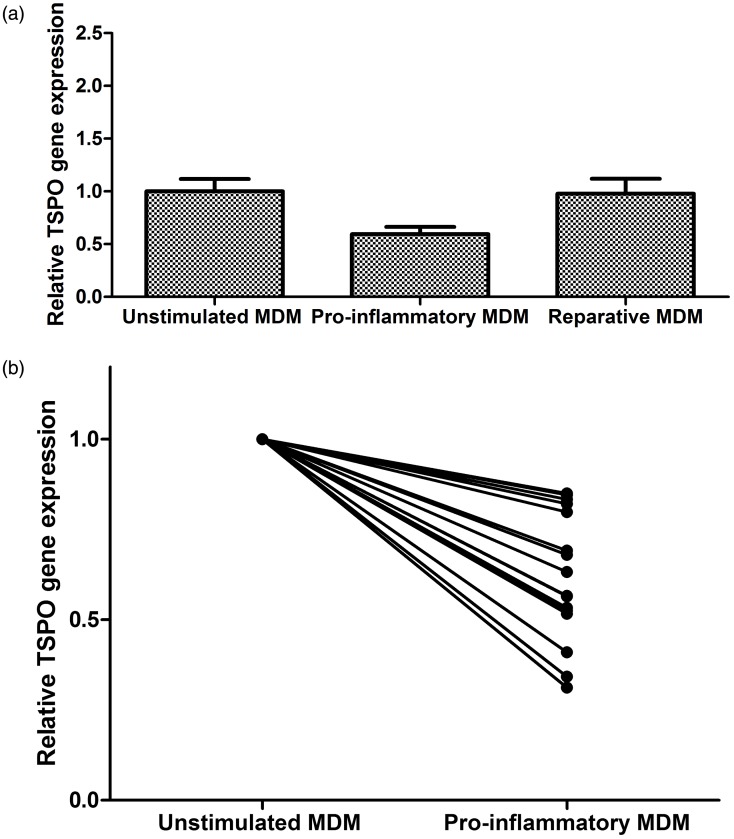
(a) Changes in TSPO gene expression of human monocyte-derived macrophages (MDM) under stimulated conditions relative to unstimulated conditions (n = 15, ANOVA, *p* = 0.02). (b) Plot comparing individual MDM TSPO gene expression responses to pro-inflammatory stimulatory conditions (n = 15, paired *t* test *p* < 0.0001).

*TSPO* gene expression also did not increase in MDM (n = 15) with stimulation. In fact, IFN-γ/LPS stimulation was associated with a decrease in *TSPO* gene expression (one way ANOVA, *p* = 0.02, Figure 3(a)). Relative to unstimulated cells, IFN-γ/LPS-stimulated MDM showed a mean expression level of 0.60 (95% CI 0.45–0.74); each of 15 samples showed a reduction (Figure 3(b)). IL4/IL13 stimulation did not affect *TSPO* gene expression in MDM (0.98-fold change [CI 0.67–1.29]).

### TSPO gene expression increases in activated primary rodent myeloid cells

**Figure 4.**
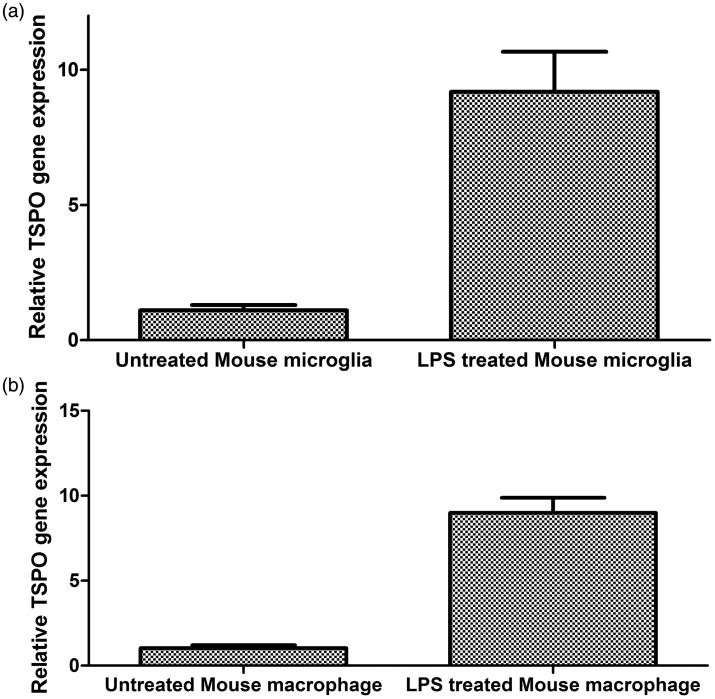
Changes in TSPO gene expression of rodent myeloid cells under pro-inflammatory conditions relative to unstimulated conditions. (a) Mouse microglia (n = 5, paired *t* test *p* < 0.0007). (b) Mouse macrophages (n = 3, paired *t* test *p* < 0.0009).

Because the rodent myeloid cell has been reported previously to show an increase in *TSPO* gene expression after pro-inflammatory activation, we repeated the same experiments with primary rodent myeloid cells. In primary rodent macrophages, LPS stimulation increased *TSPO* gene expression by approximately 9-fold (*t* test, *p* = 0.0009, Figure 4). In primary rodent microglia, LPS stimulation also increased *TSPO* gene expression by approximately 9-fold (t test, *p* = 0.0007, Figure 4).

### Radioligand binding in myeloid cells upon cellular activation

**Figure 5.**
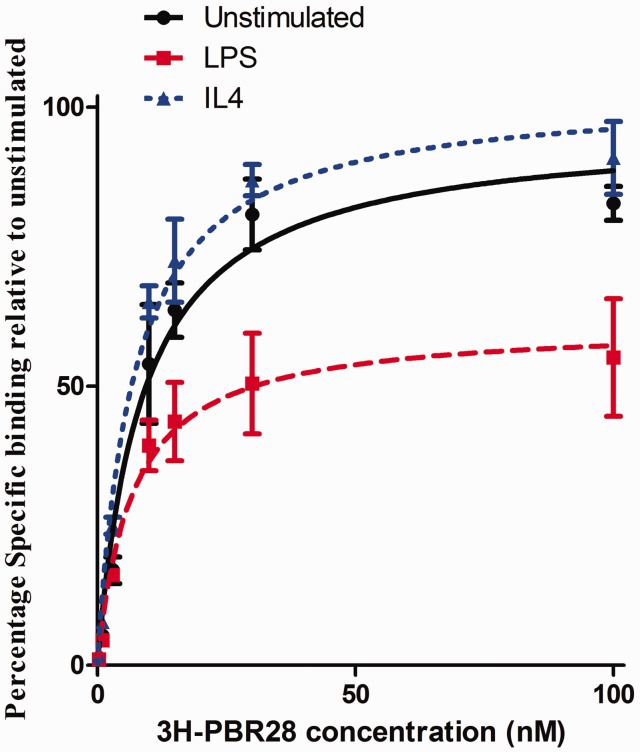
Effect of stimulatory conditions in human monocyte derived macrophages (MDM) on ^3^H-PBR28 radioligand binding parameters B_max_ and K_d_. (K_d_, n = 3, F test, *p* = 0.65. B_max_, n = 3, F test, *p* ≤ 0.0001). For each condition, the plot represents the mean of three independent donors. Each concentration was assayed in triplicate for each donor.

Radioligand binding experiments were performed in MDM. As the rs6971 polymorphism alters binding affinity of ^3^H-PBR28 for TSPO,^33^ only cells from donors homozygous for the common allele were included. Under basal conditions in MDM, ^3^H-PBR28 bound TSPO with an affinity of 8.75 nM and a B_max_ of 33,931 fmol binding sites/mg protein. Under pro-inflammatory or reparative conditions, binding affinity did not significantly change (*p* = 0.65, Figure 5). However, we observed a reduction in B_max_ under pro-inflammatory conditions, relative to the unstimulated condition (0.61-fold change [95% CI 0.49–0.73], *p* < 0.0001, Figure 5)). There was no change in B_max_ (1.03-fold change [95% CI 0.92–1.13], *p* = 0.43) with IL4 stimulation relative to the unstimulated condition. TSPO protein expression measured by Western blotting in MDM under pro-inflammatory conditions was consistent with radioligand binding data (0.79 fold change in pro-inflammatory relative to the unstimulated condition, n = 2). Radioligand binding was not performed in HAM because of the large protein requirement. As with MDM, TSPO protein expression in pro-inflammatory HAM was also reduced relative to the unstimulated condition (0.71 fold change, n = 3).

## Discussion

Here, we show that TSPO expression in primary human macrophages and microglia does not increase following pro-inflammatory activation. In fact, human macrophages show a substantial and similar (approximately 40%) reduction in both *TSPO* gene expression and binding of the high affinity, TSPO specific radioligand, ^3^H-PBR28 under pro-inflammatory activation conditions. Interestingly, this data derived from human cells contrasts with the previous published literature that has investigated changes in TSPO using rodent cells. TSPO expression has been reported to increase in activated rodent myeloid cells, with very similar fold increases in both gene and protein expression.^13,15,21,26^ Our rodent data are consistent with the literature and showed an approximately 10-fold increase in TSPO gene expression following activation, in both macrophages and microglia.

These findings therefore have implications for the use of rodent tissue in experiments involving TSPO quantification and regulation. The fact that human primary microglia and macrophages do not respond analogously to equivalent rodent cells implies an important species difference. Rodent microglia are used extensively given the scarcity of primary human microglia; however, there are several important differences between the two cell types.^34,35^ For example, in rodent but not human microglia, transforming growth factor β1 (TGFβ_1_) inhibits IFNγ-induced microglial HLA expression.^36^ Production of nitric oxide, an important molecule in the inflammatory response, requires greater stimulation in human compared with rodent microglia, and even so human cells produce much lower levels of it.^34^ Furthermore, many of the markers with differential expression profiles in human microglia relative to human macrophages are not expressed by rodent myeloid cells.^37,38^ Our findings add to these differences and suggest that extrapolating TSPO biology from rodent to human myeloid cells should be done with extreme caution.

These data also have implications for understanding the biology of TSPO in microglia. TSPO is a highly conserved protein, richly expressed in steroidogenic and myeloid cells.^5^ Its functions in steroidogenic cells likely are related to de novo steroid synthesis, which it appears able to modulate, although it may not be a necessary part of the steroidogenic machinery.^39,40^ However, rodent microglia^26^ and human microglia (data not shown) lack CYP11A1, the enzyme necessary to synthesize steroids de novo. Therefore, it is likely that TSPO in microglia has a function unrelated to de novo steroid synthesis. Various potentially relevant alternative functional roles have been described, including roles in the regulation of the efficiency of mitochondrial oxidative phosphorylation and the production of ROS.^23,25^ The latter might contribute to potential immunoregulatory functions, e.g. through modulation of ROS activation of HIF-1α.^41^

There has been great interest in developing TSPO for imaging microglia and macrophage activation in vivo*.*^10^ For example, TSPO has been used as a PET target to assess myeloid cell activation in the brain.^42–48^ Increases in the PET signal suggesting increased TSPO density generally have been interpreted in terms of increases in TSPO expression in microglia with activation.^42,48^ However, under the assumptions that our in vitro findings reflect in vivo behaviour, our study tentatively suggests that increases in the TSPO PET specific signal in humans may reflect either local myeloid cell proliferation and or monocyte recruitment rather than microglial activation (assuming that contributions to change from other TSPO expressing cells such as astrocytes can be ignored). If so, a therapeutic intervention which alters myeloid cell phenotype from a pro-inflammatory to a reparative phenotype might not reduce the TSPO PET signal in humans, unless phenotypic modulation is also accompanied by a reduction in cell density.

These results provide robust, novel data, but have limitations. The sample sizes for the microglial preparations are small, reflecting the scarcity of the brain tissue needed. Radioligand binding assays were also not possible in this cohort because such assays require several milligrams of cell lysate. For the same reason, the sample sizes for the radioligand binding assays in macrophages were also small as large blood volumes are required, necessitating different isolation protocols. However, despite this, the LPS induced changes in macrophage ^3^H-PBR28 binding were substantial, consistent across samples, and of similar magnitude to changes in gene expression and protein expression.

The experiments described here were all performed in vitro and it is well recognised that the standard in vitro polarisation protocols, although established and widely used, do not recapitulate the complex and dynamic environment within the tissue parenchyma. Intricate immune-neural interactions significantly contribute to microglial phenotype. For example, CD200, expressed on neuronal and astrocytic cell membranes, interacts with microglial CD200R contributing to tonic inhibition of microglial activation. Loss of this inhibition induces the characteristic microglial activation cascade.^49^ Microglia also express receptors for neurotransmitters including γ-aminobutyric acid (GABA) and glutamate, providing another pathway for microglial phenotypic regulation.^1,50,51^ Caution should therefore be used when extrapolating these findings in vivo and further work will therefore examine directly how TSPO expression changes on a per cell basis with in vivo models and post mortem human tissue sections across a range of diseases. Of note, however, is that previous studies have reported unexpected, reduced TSPO radioligand binding signal in mononuclear cells isolated from patients with inflammatory diseases, relative to age-matched healthy controls. Such studies include a 35% reduction in ^3^H-PBR28 specific signal in PBMCs isolated from patients with multiple sclerosis, a 40% reduction in ^3^H-PK11195 B_max_ in monocytes isolated from patients with osteoarthritis, and an 80% reduction in ^3^H-PK11195 B_max_ in macrophages isolated from broncho-alveolar lavage of patients with lung fibrosis.^52–54^ Furthermore, recent studies in conditions characterised by microglial activation but without peripheral monocyte recruitment (such as alcohol withdrawal and schizophrenia) have not demonstrated the expected increase in TSPO PET signal.^55,56^ These unexpected findings merit reconsideration in light of the data we present here.

Likewise, data showing a robust increase in CNS ^11^C-PBR28 PET signal following systemic LPS administration to both humans and nonhuman primates may need to be reassessed.^57,58^ In these experiments, the increased TSPO PET signal was interpreted as evidence of microglial activation. Our data does not refute the hypothesis that systemic LPS activates microglia. However, it does suggest that the apparent increase in modelled CNS TSPO radioligand uptake following systemic LPS administration might not be due to greater microglial activation: other possible causes need to be considered also. For example, adherence of circulating leucocytes to the vascular endothelium and recruitment of peripheral monocytes into the parenchyma are both rapid processes that would increase the TSPO PET signal in the CNS.^59,60^ Endothelial cells also express both TSPO and Toll-like receptor 4 (TLR4, an LPS receptor). An increase in endothelial TSPO expression would also increase the CNS TSPO PET signal.

In conclusion, while TSPO expression increases in primary rodent microglia and macrophages with pro-inflammatory activation, this does not occur in primary human microglia. Futhermore, TSPO reduces upon activation of primary human macrophages. This observation has implications for understanding TSPO biology in humans, and for interpreting differences in TSPO PET binding data in clinical populations.

## Data

Data underpinning this study is available at zenodo (https://doi.org/10.5281/zenodo.556104) and is made openly available via a Creative Commons CC-BY license.
